# Macrophage Migration Inhibitory Factor Triggers Inflammatory Responses During Very Virulent Infectious Bursal Disease Virus Infection

**DOI:** 10.3389/fmicb.2019.02225

**Published:** 2019-10-01

**Authors:** Aijing Liu, Hui Li, Xiaole Qi, Qi Wang, Bo Yang, Tiantian Wu, Nana Yan, Yue Li, Qing Pan, Yulong Gao, Li Gao, Changjun Liu, Yanping Zhang, Hongyu Cui, Kai Li, Yongqiang Wang, Xiaomei Wang

**Affiliations:** ^1^Division of Avian Infectious Diseases, State Key Laboratory of Veterinary Biotechnology, Harbin Veterinary Research Institute, Chinese Academy of Agricultural Sciences, Harbin, China; ^2^Jiangsu Co-innovation Center for Prevention and Control of Important Animal Infectious Diseases and Zoonoses, Yangzhou, China

**Keywords:** vvIBDV, inflammation, MIF, macrophage, proinflammatory cytokines

## Abstract

Infectious bursal disease (IBD) is one of the main threats to the poultry industry worldwide. In China, very virulent IBD virus (vvIBDV) is the main prevalent virus strain, causing inflammation, immunosuppression, and high mortality in young chickens. To determine whether this acute inflammation can trigger lesions or even death in chickens, it is important to study the mechanism of vvIBDV pathogenicity. Thus, in the current study, we investigated the inflammation response, bursal lesions, and mortality in chickens caused by vvIBDV at different time points postinfection. Results showed an upregulation of proinflammatory cytokines, including interleukin-1β and interleukin-18, and macrophage infiltration in bursa in response to vvIBDV infection. High-throughput proteomic sequencing based on isobaric tags for relative and absolute quantitation showed that chicken macrophage migration inhibitory factor (chMIF) was upregulated uniquely in primary bursal cells infected with vvIBDV compared with infection by nonpathogenic attenuated IBDV. We confirmed that chMIF was upregulated by vvIBDV infection both *in vivo* and *in vitro*. Moreover, chMIF was extracellularly secreted by infected DT40 and primary bursal cells. Further experiments revealed that the secreted chMIF could induce migration of peripheral blood mononuclear cells and promote transcription of proinflammatory cytokines in chicken primary macrophages. Notably, these effects of chMIF could be reduced by using an MIF specific inhibitor. Thus, our study elucidates critical molecular determinants underlying vvIBDV-mediated initiation of acute inflammation, which might be pivotal to understand the mechanism of vvIBDV pathogenicity.

## Introduction

Infectious bursal disease virus (IBDV) causes highly contagious and immunosuppressive disease in young chickens, resulting in great losses in the poultry industry worldwide (Lasher and Davis, 1997; van den Berg et al., 2000). IBDV is a typical non-enveloped, double-stranded RNA, icosahedral virus belonging to the genus *Avibirnavirus* of the family Birnaviridae (Azad et al., 1985). Since its first identification in 1957 (Winterfield and Hitchner, 1962), variable virulent strains of IBDV have arisen after the development of two large mutations. Depending on the antigenicity and pathogenicity in chickens, IBDV is now classified as follows: classical IBDV (vIBDV), antigenic variant IBDV (avIBDV) (Winterfield and Hitchner, 1962), intermediate IBDV, very virulent IBDV (vvIBDV) (Chettle et al., 1989; Annamalai et al., 2016), and attenuated IBDV (aIBDV) virulent strains (Zhang et al., 1997). The pathological symptoms of IBDV mainly include bursa of Fabricius (BF) destruction and immunosuppression (Hirai and Shimakura, 1974; Lasher and Davis, 1997). However, the pathogenic mechanism underlying IBDV infection is not completely clear. Recent studies have provided evidence that vIBDV and vvIBDV infections also lead to inflammation, manifested as upregulation of proinflammatory cytokines and chemokines, as well as migration of inflammatory cells (Sharma et al., 1989; Abdel-Alim and Saif, 2001; Eldaghayes et al., 2006; Ruby et al., 2006; Ou et al., 2017; Wang et al., 2019). Infection of 4-week-old chicken with 2 × 10^3^ EID_50_ of vvIBDV per chicken was reported to result in 64% mortality (Wang et al., 2004).

The chicken macrophage migration inhibitory factor (chMIF) was identified in early embryo eye lens in 1993 (Wistow et al., 1993). ChMIF is composed of 115 amino acids and shows 71% identity with the human and murine MIFs (Kim et al., 2010). ChMIF was able to inhibit random migration of chicken macrophages in a dose-dependent manner, similar to human MIF. Also, chMIF can enhance the levels of interleukin (IL)-1β, inducible nitric oxide synthase (iNOS), and interferon (IFN)-γ during peripheral blood mononuclear cell (PBMC) stimulation with lipopolysaccharide (LPS). Once stimulated by mitogen, the expression level of IFN-γ in lymphocyte could be upregulated *via* chMIF, and the mRNA levels of IL-4 and IL-13 were reduced. Similar to mammalian MIF, chMIF can mediate inflammatory reactions during antigenic stimulation (Kim et al., 2010). Both chicken and parasite MIF molecules can bind to chicken macrophages *via* the surface receptor chCD74 (Kim et al., 2014). However, the exact function of chMIF in virus infection has remained partially unclear.

The current study was designed to investigate whether vvIBDV-induced inflammation performs antiviral function or excess inflammation triggers lesions or even death in chickens infected with vvIBDV. To uncover the relationship between inflammation and bursal lesions caused by vvIBDV infection, the different host protein changes in primary bursal cell infected with vvIBDV or aIBDV were observed using high-throughput proteomic sequencing based on isobaric tags for relative and absolute quantitation (iTRAQ) technology. Based on our results, we further characterized the modulation of MIF, which was found to be upregulated in vvIBDV-infected primary bursal cells. The role of chMIF in proinflammatory cytokine production triggered by virus infection was also explored. The results of this study are expected to significantly contribute toward understanding the mechanism of vvIBDV pathogenicity.

## Materials and Methods

### Viruses and Reagents

The vvIBDV strain Gx was previously identified and has been preserved in our laboratory (48). The viral loads of Gx strain were detected by performing 50% egg lethal dosage (ELD_50_). Briefly, the vvIBDV was diluted by PBS and then inoculated on the chorioallantoic membrane of 9-day-old SPF egg (purchased from Harbin Veterinary Research Institute). The mortality of eggs was observed daily in 7 days postinfection (d.p.i.).

The yeast-expressed recombinant MIF protein was purchased from Abcam (ab222155). Iodo-6-phenylpyrimidine (4-IPP) was purchased from Sigma. 4,5-Dihydro-3-(4-hydroxyphenyl)-5-isoxazoleacetic acid methyl ester (ISO-1) and iguratimod were derived from Selleck Chemicals (United States). Rabbit anti-MIF antibody (short peptide antigen) was custom-made by Nanjing Gen Script Company.

### Animals

All animal experiments were approved by the Committee on the Ethics of Animal Experiments at the Harbin Veterinary Research Institute (Harbin, China), Chinese Academy of Agricultural Sciences, and performed in accordance with the Guidelines for Experimental Animals of the Ministry of Science and Technology (Beijing, China). All chickens were cared for in accordance with humane procedures. The animal ethics committee approval number is Heilongjiang-SYNK-2017-009. SPF White Leghorn chickens were derived from Harbin Veterinary Research Institute (China). To establish a chicken model of vvIBDV and aIBDV infections, 140 3-week-old specific-pathogen-free (SPF) chickens were randomly distributed into two groups and challenged intranasally with a total volume of 200 μl vvIBDV (1 × 10^2^ ELD_50_, < 20% lethal to chicken for observation as long as 7 days) or phosphate-buffered saline (PBS; pH 7.0) per chicken; PBS was used for the viral dilutions. Serum and bursal samples were collected for the subsequent experiments.

### Cell Cultures

DT40 cells (chicken lymphoma cell line) were a generous gift from Prof. Venugopal Nair at The Pirbright Institute. These cells were cultured at 37°C in a 5% CO_2_ atmosphere in Roswell Park Memorial Institute (RPMI)-1640 medium (Gibco, United States) and supplemented with 10% fetal bovine serum (FBS), 2% chicken serum (Sigma), 1% L-glutamine (Gibco), and 0.1% β-mercaptoethanol.

Peripheral blood mononuclear cells were obtained from the whole blood of SPF chickens and cultured at 37°C in a 5% CO_2_ atmosphere in RPMI-1640 medium (Gibco) supplemented with 10% FBS. Chicken whole blood was added above the liquid level of Histopaque 1119 (Sigma) and centrifuged at 2,000 rpm for 25 min. Isolated cells at the interface of the medium and Histopaque 1119 were washed by PBS for three times and resuspended by RPMI-1640. Cells were then added to upper chamber for Transwell assays.

Primary bursal cells were obtained from the BF of 3-week-old SPF chickens and cultured at 42°C in 5% CO_2_ atmosphere in Iscove’s modified Dulbecco’s medium (IMDM; Hyclone, United States) supplemented with 10% FBS (Sigma), 3% chick embryo fibroblast supernatant, 2% chicken serum (Sigma), 1% l-glutamine (Gibco), 1% insulin, transferrin, selenium solution (Sigma), and 0.1% β-mercaptoethanol (Sigma). Cells were separated according to previously described methods (Dulwich et al., 2018). Briefly, bursas were harvested and washed with sterile PBS and digested by collagenase D (Sigma) in Hanks balanced salt solution supplemented with calcium (HBSS, Gibco). Then, the digested tissue was passed through a 40-μM Falcon cell strainer (Thermo Fisher Scientific) into HBSS (Gibco). The mixture was resuspended by complete medium for bursa cells (as described above) and centrifuged over Histopaque 1083 (Sigma) at 2,000 rpm for 25 min. Cells at the interface of the medium and Histopaque 1083 were harvested and washed three times with RPMI-1640 with 10% FBS (Gibco).

Primary chicken macrophages were prepared from the bone marrow of 3-week-old SPF chickens and cultured at 38.5°C in a 5% CO_2_ atmosphere in RPMI-1640 medium supplemented with 10% tryptose phosphate broth (Sigma), 5% FBS, 5% chicken serum, 1% sodium pyruvate (Gibco), and 0.1% β-mercaptoethanol. Briefly, femurs were collected in sterile conditions, and the ends of femurs were removed. Then bone marrow was flushed out and passed through a 40-μM Falcon cell strainer into RPMI-1640 medium with slight grinding. The mixture was centrifuged over Histopaque 1083 (Sigma) at 2,000 rpm for 25 min. Cells at the interface of the medium and Histopaque 1083 were harvested and washed with RPMI-1640 with 10% FBS (Gibco) for three times. Cells were maintained, supernatants were removed, and adherent cells were washed twice with RPMI-1640 to remove nonadherent and semiadherent cells. After being maintained for 6 days, cells were collected and identified by flow cytometry (BD Accuri C6 plus) using mouse anti-chicken monocyte/macrophage antibody KUL01-PE (Southern Biotech) to evaluate the cell surface expression of markers typically expressed on chicken macrophage by flow cytometry and then used for other experiments (Feng et al., 2017).

### Isobaric Tag for Relative and Absolute Quantitation

B lymphocytes were infected with 0.1 multiplicity of infection (MOI) vvIBDV or aIBDV. Cell samples were sent to LC Sciences (Hangzhou, China) for iTRAQ detection, including protein extraction, digestion, desalting, iTRAQ labeling and fractionation, and data analysis.

### Histopathology

Bursal samples from the chickens were fixed in 10% formalin for 48 h at room temperature (RT) and then dehydrated, embedded in paraffin wax, and cut into 5-μm-thick sections. The sections were stained with hematoxylin and eosin (H&E) and examined using light microscopy.

### RNA Extraction and Quantitative Polymerase Chain Reaction

Specific primers and TaqMan probes for chicken 28S rRNA (Giotis et al., 2015), IL-1β, IL-18 (Yasmin et al., 2015), chMIF (Table 1), and IBDV virus load (Wang et al., 2009) were synthesized by Invitrogen (China). Primer and probe sequences used for quantitative polymerase chain reaction (qPCR) analysis were described in Table 1. Total RNA was extracted using the RNeasy mini kit (Qiagen, Germany), and 2 μg RNA was reverse transcribed to cDNA using the ReverTra Ace qPCR RT Master Mix with gDNA Remover (Toyobo, Japan) in a 20-μl reaction mixture. The cDNA was analyzed by qPCR performed using Premix Ex Taq (Probe qPCR) (Takara, Japan). The qPCR was performed under the following cycling conditions: initial denaturation at 95°C for 30 s, followed by 45 cycles each of 95°C for 5 s and 60°C for 20 s. All controls and infected samples were examined in triplicate on the same plate. The cDNA copies were normalized to 28S cDNA copies measured from the same samples. The 2^–ΔΔ*Ct*^ method was used for data analysis and relative quantification.

**TABLE 1:** Primer and probe sequences used for quantitative polymerase chain reaction (PCR) analysis.

**RNA target**	**Probe/Primer sequence (5′-3′)**
28S	Probe: (FAM-AGGACCGCTACGGACCTCCACCA- TAMRA) F: GGCGAAGCCAGAGGAAACT R: GACGACCGATTTGCACGTC
Infectious bursal disease virus (IBDV) copies	Probe: (FAM- CGGCGTCCATTCCGGACGAC- TAMRA) F: GAGCCTTCTGATGCCAACAAC R: CAAATTGTAGGTCGAGGTCTCTGA
Interleukin (IL)-1β	Probe: (FAM-CACACTGCAGCTGGA-TAMRA) F: GCTCTACATGTCGTGTGTGATGAG R: TGTCGATGTCCCGCATGA
Interleukin (IL)-18	Probe: (FAM-CACACTGCAGCTGGA -TAMRA) F: AGGTGAAATCTGGCAGTGGAAT R: ACCTGGACGCTGAATGCAA
Macrophage migration inhibitory factor (MIF)	Probe: (FAM-CCTTCAGCAGGGATG-TAMRA) F: GTGCGATATGATTGCGAAGC R: AGGAGCCATCCATCTGTGAGT

### Enzyme-Linked Immunosorbent Assay

The MIF concentrations in the cell culture medium were tested using an enzyme-linked immunosorbent assay (ELISA) kit from ABclonal (United States), following the manufacturers’ instructions. The detection limits of the kit were 500 to 10,000 pg/ml. All of the samples were diluted 10-folds to be ensured within the limitations.

### Chemotaxis Assays

Isolated PBMCs were seeded at 10^4^ cells/200 μl/well in serum-free RPMI 1640 medium in the upper Transwell chamber (5-μm polycarbonate membrane; Costar) of a 24-well plate. The cell culture medium of DT40 or bursal cells infected with IBDV; 4- IPP-, ISO- 1-, iguratimod-, or DMSO (Ameresco)-treated cell supernatant, and different concentrations of MIF (0, 1, 10, 50, 100, 1,000, 2,000, 3,000, 4,000, or 5,000 ng/ml in serum-free RPMI 1640) were added to the lower chamber. The cells were added to the top chamber and incubated at 37°C for 2 h. The cells in the upper chamber were removed with a sterile cotton swab, and the cells in the lower chamber were carefully preserved and fixed with absolute methanol solution at RT. The transmigrated cells in the lower chamber were stained with 4′-6-diamidino-2-phenylindole (DAPI; Beyotime Biotechnology, China), and images were captured with an EVOS F1 inverted fluorescence microscope (AMG, United States). The cell numbers were calculated with the ImageJ software.

### Cell Counting Kit-8

Cell proliferation was evaluated using Cell Counting Kit 8 (CCK-8; Dojindo, Tokyo, Japan) according to the manufacturer’s instructions. The absorbance value for each well was measured at 450 nm with a Multiskan FC microplate reader.

### Statistical Analysis

Statistical analyses were performed with the one-way analysis of variance (ANOVA). A *P*-value less than 0.05 was considered statistically significant. Data are reported as mean ± standard deviation.

## Results

### VvIBDV Infection Led to Inflammation of the Bursa and Mortality in SPF Chickens

**FIGURE 1:**
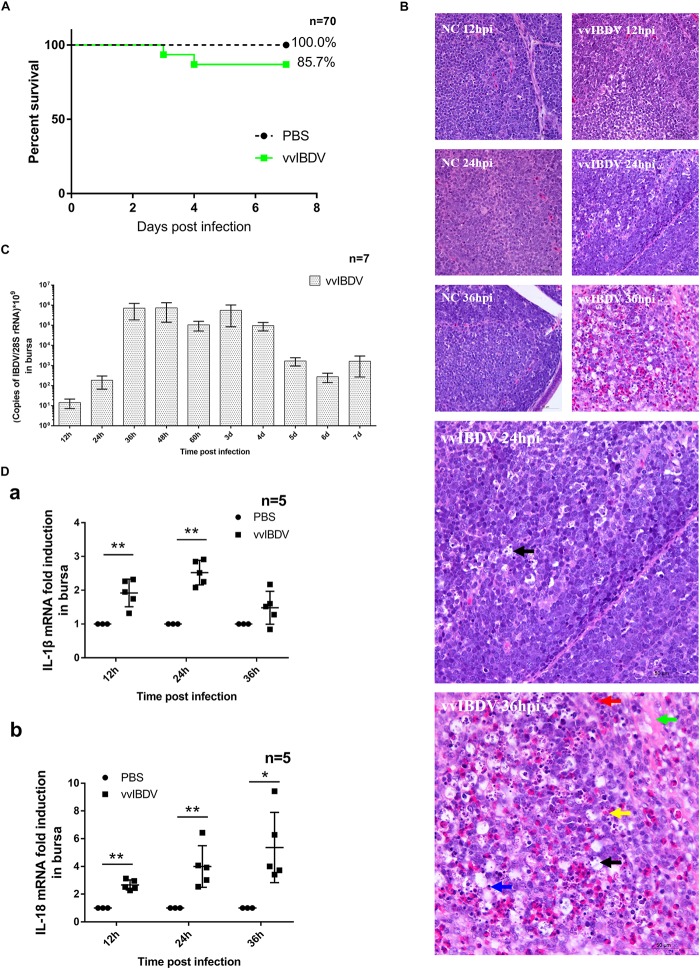
Very virulent infectious bursal disease virus (vvIBDV) infection led to inflammation of the bursa and mortality in specific-pathogen-free (SPF) chickens. Three-week-old SPF chickens were infected with 1 × 10^2^ ELD_50_ vvIBDV or were mock infected with phosphate-buffered saline (PBS) alone. The chickens were sacrificed at the following time points: 12 h, 24 h, 36 h, 48 h, 60 h, 3 days, 4 days, 5 days, 6 days, and 7 days. **(A)** Survival curves were based on the mortality recorded at each time point. **(B)** For histopathological analysis, bursal samples from vvIBDV-infected chickens stained with H&E were examined using light microscopy. In the vvIBDV infection group, macrophage proliferation (black arrow) was observed in individual follicles at 24 h.p.i. as well as slight hemorrhage (red arrow), depletion of lymphocytes (blue arrow), lesions in the bursal follicles (green arrow), proliferation of reticulum cells and macrophages (black arrow), and infiltration of inflammatory cells (yellow arrow) at 36 h.p.i. **(C)** VvIBDV replication in bursa samples. **(D)** The transcription levels of host inflammatory cytokines IL-1β and IL-18 were measured by quantitative polymerase chain reaction (qPCR). ^∗^*P* < 0.05 and ^∗∗^*P* < 0.01 compared with control groups.

To investigate the pathogenic mechanism underlying vvIBDV infection, 3-week-old SPF chickens were infected with vvIBDV (1 × 10^2^ ELD_50_) or were mock-infected with PBS alone. All chickens were observed for 7 d.p.i. They were sacrificed at the following time points: 12 h, 24 h, 36 h, 48 h, 60 h, 3 days, 4 days, 5 days, 6 days, and 7 days (seven chickens per group). The mortality for each group was recorded at each time point, and survival curves were drawn based on these data (Figure 1A). Some chickens in the vvIBDV infection group died within 4 d.p.i. (two died at 3 d.p.i. and the other two died in 4 d.p.i.), with a mortality rate of 14.3%. However, no mortality or clinical signs were found in the PBS control group.

Bursas were collected for analysis of histological lesions and detecting virus replication. In the vvIBDV infection group, histological analysis showed macrophage proliferation (black arrow) in individual follicles at 24 h postinfection (h.p.i.) and slight hemorrhage (red arrow), depletion of lymphocytes (blue arrow), lesions in the bursal follicles (green arrow), proliferation of reticulum cells and macrophages (black arrow), and infiltration of inflammatory cells (yellow arrow) at 36 h.p.i. These observations indicated that inflammation was induced by vvIBDV infection (Figure 1B). No obvious pathological change was observed in the PBS control groups (Figure 1B). Virus was verified to replicate effectively in bursa, peaking at 36 h.p.i. until 4 d.p.i. (Figure 1C). Taken together, our findings indicate that vvIBDV infection caused bursal lesions and mortality in SPF chickens and induced inflammation in the bursa.

To further understand the histological inflammatory response in the bursa, we assessed whether proinflammatory cytokines IL-1β and IL-18 were triggered during vvIBDV infection. The transcription levels of IL-1β (at 12 and 24 h.p.i.) and IL-18 (from 12 to 36 h.p.i.) were significantly (*P* < 0.05) increased in the vvIBDV infection group compared with the corresponding control groups (Figure 1D). Thus, the increased levels of the proinflammatory cytokines suggested that vvIBDV infection boosted the mRNA level of major inflammatory cytokines IL-1β and IL-18.

### ChMIF Was Upregulated in VvIBDV Infection Both *in vivo* and *in vitro*

**FIGURE 2:**
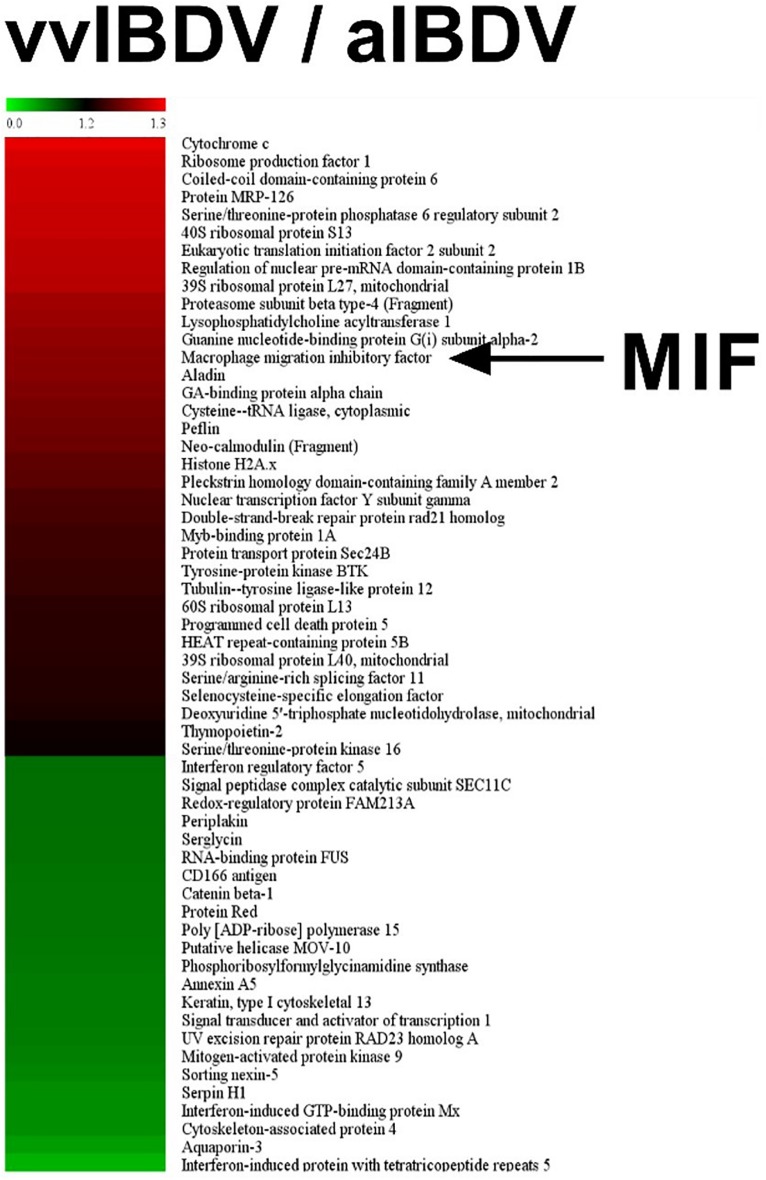
iTRAQ LC-MS/MS was conducted on very virulent infectious bursal disease virus (vvIBDV)- and attenuated IBDV (aIBDV)-infected B lymphocyte samples at 36 h.p.i. The expression level of proinflammatory factor macrophage migration inhibitory factor (MIF) was found to be higher in the vvIBDV-infected group than in the aIBDV-infected group (1.26-fold).

**FIGURE 3:**
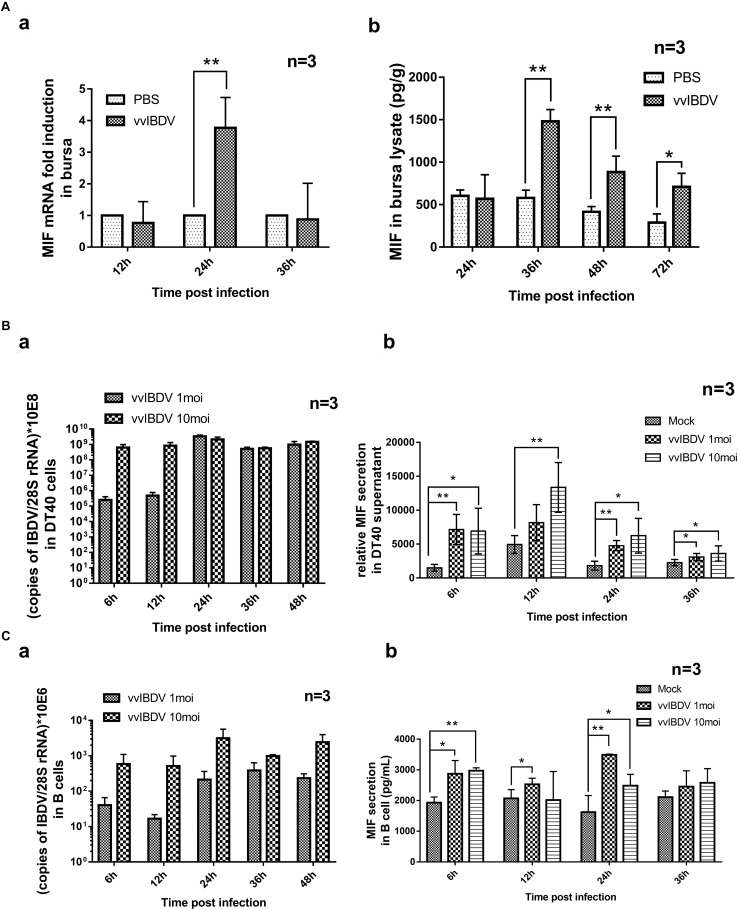
Chicken macrophage migration inhibitory factor (chMIF) was upregulated in response to very virulent infectious bursal disease virus (vvIBDV) infection both *in vivo* and *in vitro.* **(A)** The bursas from vvIBDV-infected animals were analyzed for chMIF transcription or expression analysis. **(a)** The transcription level of chMIF at 24 h.p.i. **(b)** Enzyme-linked immunosorbent assay (ELISA) of bursal lysis samples to assess the expression of chMIF in response to IBDV infection. ^∗^*P* < 0.05 and ^∗∗^*P* < 0.01 compared with control groups. **(B)** DT40 cells were infected with 1 and 10 multiplicity of infection (MOI) of vvIBDV, and cells and supernatants were collected for quantitative polymerase chain reaction (qPCR) and ELISA. **(a)** Replication of vvIBDV in DT40 cells. **(b)** Secretion of chMIF in vvIBDV-infected groups, as detected by ELISA. **(C)** Bursal cells were infected with 1 and 10 MOI of vvIBDV, and cells and supernatants were collected for qPCR and ELISA. **(a)** Replication of vvIBDV in primary bursal cells. **(b)** Secretion of chMIF in vvIBDV-infected groups, as detected by ELISA. ^∗^*P* < 0.05 and ^∗∗^*P* < 0.01 compared with control groups.

To observe the expression of inflammation-associated proteins in IBDV-infected target cells, iTRAQ LC-MS/MS was conducted on vvIBDV- and aIBDV-infected B lymphocyte samples at 36 h.p.i. In total, 277 proteins were identified and quantified to be the differentially expressed proteins between vvIBDV- and aIBDV-infected samples. After data filtering about vvIBDV relative to aIBDV infection samples (≥1.2-fold change for upregulation or ≤0.8-fold change for downregulation), 58 significant differentially expressed proteins (35 upregulated and 23 downregulated) were observed (Figure 2). Among these results, three inflammation-associated proteins were identified (two for upregulation and one for downregulation) based on the GO analysis of biological process. The expression level of proinflammatory factors MIF and protein MRP-126 in the vvIBDV-infected group was higher than that in the aIBDV-infected group. The inflammatory suppression proteins selenoprotein H were lower in the vvIBDV-infected samples. Because the exact function of protein MRP-126 was poorly studied, we chose the proinflammatory cytokine MIF to investigate the process of vvIBDV-induced inflammation. High levels of MIF are indicative of autoimmune diseases and severe inflammatory states (Mizue et al., 2005). For confirming the iTRAQ data, the bursas in the animal experiments mentioned above were sampled for chMIF mRNA or protein levels. The mRNA level of chMIF was significantly (*P* < 0.05) promoted by vvIBDV infection compared with the PBS control at 24 h.p.i. (Figure 3Aa). Furthermore, ELISA indicated that the protein load of chMIF in the vvIBDV infection group significantly (*P* < 0.05) and continually increased from 36 to 72 h.p.i. (Figure 3Ab). There were no obvious changes at other time points or in the PBS control. Thus, vvIBDV infection both promoted the transcription and increased the protein levels of the host MIF *in vivo*.

To elucidate whether chMIF increased after vvIBDV infection *in vitro*, DT40 cells and primary bursal cells were infected at 1 or 10 MOI. The cells and supernatants were collected for qPCR and ELISA. The vvIBDV was effectively replicated in DT40 (Figure 3Ba) and primary bursal cells (Figure 3Ca). ChMIF secretion upon vvIBDV infection significantly increased (*P* < 0.05) both in the infected DT40 (6–36 h.p.i.) and bursal cell supernatants (6–24 h.p.i.; Figures 3Bb,Cb) compared with the mock infection. These data together indicated that chMIF was upregulated in vvIBDV infection both *in vivo* and *in vitro*. Moreover, the *in vivo* induction of MIF by vvIBDV occurred earlier than the upregulation of IL-1β/IL-18 transcription and expression.

### Migration of PBMCs That Was Mediated by chMIF and vvIBDV-Infected DT40 Cell Supernatants

**FIGURE 4:**
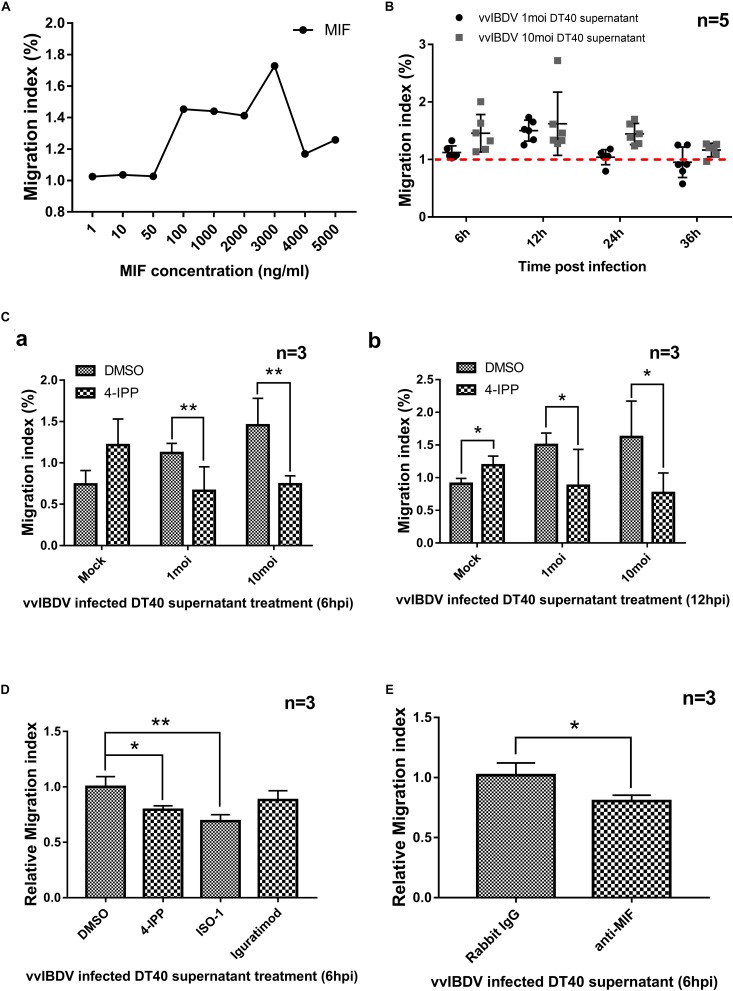
Migration of peripheral blood mononuclear cells (PBMCs) was mediated by chicken macrophage migration inhibitory factor (chMIF) and very virulent infectious bursal disease virus (vvIBDV)-infected DT40 cell supernatants. Transwell migration assays were conducted using PBMCs seeded in the upper chamber and cell culture medium of vvIBDV-infected DT40 cells and different concentrations of MIF added to the lower chamber. **(A)** The PBMC migration index (number of migrating cells in the experimental group/number of migrating cells in the negative control), determined at different concentrations (0, 1, 10, 50, 100, 1,000, 2,000, 3,000, 4,000, or 5,000 ng/ml) of MIF *via* Transwell experiments. **(B)** DT40 cells were infected with 1 multiplicity of infection (MOI) and 10 MOI of vvIBDV; supernatant was collected and added into the lower Transwell chamber, and the PBMC migration index was noted. **(C)** The DT40 supernatants[1 or 10 MOI vvIBDV infected, 6 12 h.p.i. **(a)** and 12 h.p.i. **(b)**] were incubated with 200 μm iodo-6-phenylpyrimidine (4-IPP) (MIF antagonist) or DMSO for 4 h at room temperature and then analyzed for the PBMC migration index. **(D)** A total of 10 μM of 4-IPP/ISO-1/iguratimod were incubated with vvIBDV-infected DT40 supernatant (6 h.p.i.) or equal volume DMSO for 4 h at room temperature. Then the supernatants were used for Transwell assay to detect the PBMC migration. Relative migration index was calculated by migration index of inhibitor treated groups/migration index of DMSO-treated group. The promotion of PBMC migration by IBDV infection was significantly inhibited by 4-IPP and ISO-1, except iguratimod. **(E)** Anti-MIF antibody (or rabbit IgG) was incubated with vvIBDV-infected DT40 supernatant (6 h.p.i.) for 10 h at 4°C. The supernatants were used for Transwell assay, and the results were calculated as described above. The anti-MIF antibody was able to inhibit the promotion of PBMC migration by IBDV infection. ^∗^*P* < 0.05 and ^∗∗^*P* < 0.01 compared with control groups.

To further investigate the proinflammatory effect of chMIF, the PBMC migration index (number of migrating cells in the experimental group/number of migrating cells in the negative control) was determined at different concentrations (0, 1, 10, 50, 100, 1,000, 2,000, 3,000, 4,000, or 5,000 ng/ml) of MIF *via* a Transwell experiment, as described previously (Bernhagen et al., 2007). ChMIF triggered the migration of PBMCs from 100 to 4,000 ng/ml compared with the absence of MIF; the internalization effect vanished at higher concentrations (5,000 ng/ml) (Figure 4A).

Chicken macrophage migration inhibitory factor was secreted upon vvIBDV infection of DT40 and bursal cells; therefore, we investigated whether the supernatant of infected cells could promote PBMC migration. DT40 cells were infected with 1 or 10 MOI of vvIBDV, and supernatants were collected at the indicated time points and added into the lower Transwell chamber to detect the migration of PBMCs. The supernatants collected at 6, 12, and 48 h.p.i. for 1 MOI and those collected at 12, 24, and 48 h.p.i. for 10 MOI vvIBDV infection could increase the PBMC migration index compared with the mock infection groups (Figure 4B).

To clarify if the migration promotion of vvIBDV-infected cell supernatant was directly caused by MIF, the DT40 supernatant (1/10 MOI vvIBDV infected, 6 and 12 h.p.i.) was incubated with 200 μM 4-IPP (Varinelli et al., 2015), an MIF antagonist, or DMSO for 4 h at room temperature and then subjected to the Transwell experiments as described above. The migration index of vvIBDV-infected supernatants, both at 6 (Figure 4Ca) and 12 h.p.i. (Figure 4Cb), was significantly reduced by 4-IPP treatment. These results indicated that MIF was directly involved in the vvIBDV-infected DT40 supernatant-induced PBMC migration.

To further confirm the MIF-promoted PBMC migration by vvIBDV infection, MIF antibody and other MIF inhibitors (ISO-1 and iguratimod) were used. A total of 10 μM of 4-IPP/ISO-1/iguratimod were incubated with vvIBDV-infected DT40 supernatant (6 h.p.i.) or equal volume DMSO for 4 h at RT. Then the supernatants were used for Transwell assay to detect the PBMC migration. Relative migration index was calculated by migration index of inhibitor treated groups/migration index of DMSO-treated group. The results showed that the promotion of PBMC migration by IBDV infection was significantly inhibited by 4-IPP and ISO-1, except iguratimod (Figure 4D).

In another experiment, 50 μg/ml anti-MIF antibody (or rabbit IgG) was incubated with vvIBDV-infected DT40 supernatant (6 h.p.i.) for 10 h at 4°C. The supernatants were used for Transwell assay, and the results were calculated as described above. As Figure 4E described, the anti-MIF antibody was able to inhibit promotion of PBMC migration by IBDV infection.

Together, our results suggested that the chMIF secreted by vvIBDV-infected DT40 cells could promote the migration of PBMCs to induce the proinflammatory effect.

### Transcription of Proinflammatory Cytokines Mediated by ChMIF and vvIBDV-Infected DT40 and Bursal Cell Supernatants

**FIGURE 5:**
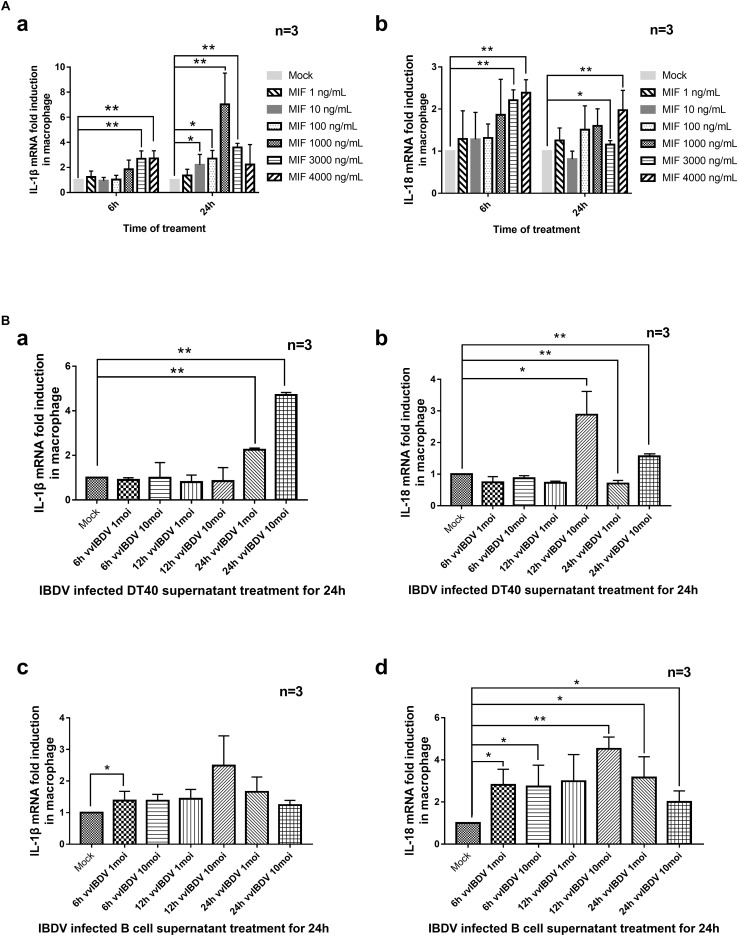
Transcription of proinflammatory cytokines was mediated by chicken macrophage migration inhibitory factor (chMIF) and very virulent infectious bursal disease virus (vvIBDV)-infected DT40 and bursal cell supernatants. **(A)** Primary macrophages were isolated and treated with different concentrations (0, 1, 10, 100, 1,000, 2,000, 3,000, or 4,000 ng/ml) of chMIF for 6 or 24 h and then subjected to quantitative polymerase chain reaction (qPCR) for analysis of transcription levels of interleukin (IL)-1β **(a)** and IL-18 **(b)**. **(B)** Primary macrophages were treated with vvIBDV or mock-infected DT40 or bursal cell supernatants for 24 h, followed by determination of the transcription levels of **(a,c)** IL-1β and **(b,d)** IL-18. ^∗^*P* < 0.05 and ^∗∗^*P* < 0.01 compared with control groups.

To assess whether MIF could induce inflammatory cytokines, primary macrophages were isolated and the purity (92.2%) was observed by flow cytometry using chicken macrophage surface marker KUL01-PE, as Supplementary Figure S1 showed. Macrophages were treated with different concentrations (0, 1, 10, 100, 1,000, 2,000, 3,000, or 4,000 ng/ml) of chMIF for 6 or 24 h. The qPCR results indicated that chMIF could increase the transcription levels of IL-1β (Figure 5Aa) and IL-18 (Figure 5Ab) after treatment for 6 h (3,000 and 4,000 ng/ml for IL-1β and IL-18, respectively) and 24 h (10–3,000 ng/ml for IL-1β and 3,000–4,000 ng/ml for IL-18).

Since chMIF was secreted by DT40 and bursal cells upon vvIBDV infection, we also investigated whether the supernatant of the infected cells could cause upregulation of IL-1β and IL-18 in response to vvIBDV infection. Primary macrophages were treated with supernatants from vvIBDV- or mock-infected DT40 or bursal cells for 24 h. The transcription levels of IL-1β (24 h.p.i.; Figure 5Ba) and IL-18 (12 and 24 h.p.i., Figure 5Bb) increased in the macrophages treated with vvIBDV-infected DT40 supernatant. The vvIBDV-infected bursal cell supernatants collected at 6 h.p.i. and 24 h.p.i. also increased the mRNA levels of IL-1β (Figure 5Bc) and IL-18 (Figure 5Bd) in macrophages.

**FIGURE 6:**
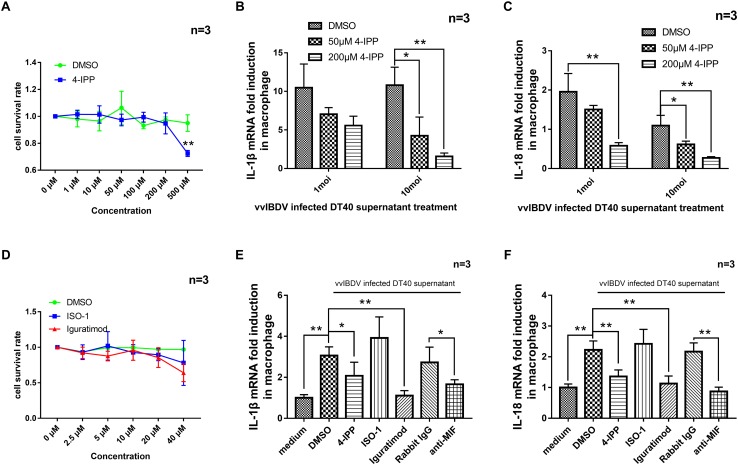
The migration of peripheral blood mononuclear cells (PBMCs) caused by very virulent infectious bursal disease virus (vvIBDV)-infected cell supernatant could be inhibited by macrophage migration inhibitory factor (MIF) inhibitor. **(A)** Different concentrations (1, 10, 50, 100, 200, and 500 μM) of iodo-6-phenylpyrimidine (4-IPP) or equal volume of DMSO solvent were added to primary macrophages, followed by assay for cell viability using CCK-8. **(B,C)** 4-IPP at concentrations of 50 or 200 μM was incubated with vvIBDV-infected cell culture medium (24 h.p.i.) and then added to macrophages for 24 h. The mRNA levels of IL-1β **(B)** and IL-18 **(C)** were then determined by quantitative polymerase chain reaction (qPCR). ^∗^*P* < 0.05 compared with control groups. **(D)** Different concentrations (2.5, 5, 10, 20, and 40 μM) of ISO-1, iguratimod, or equal volume of DMSO solvent were added to primary macrophages, followed by assay for cell viability using CCK-8. **(E,F)** A total of 20 μM of 4-IPP, 10 μM of ISO-1, iguratimod, or 50 μg/ml anti-MIF antibody were incubated with 10 multiplicity of infection (MOI) vvIBDV-infected cell culture medium (24 h.p.i.) and then added to macrophages for 24 h. The mRNA levels of IL-1β **(E)** and IL-18 **(F)** were then determined by qPCR. ^∗^*P* < 0.05 and ^∗∗^*P* < 0.01 compared with control groups.

Next, we added different concentrations (1, 10, 50, 100, 200, and 500 μM) of 4-IPP or equal volume of DMSO solvent to primary macrophage for CCK-8 experiment to detect the cytotoxicity of 4-IPP. A concentration of less than or equal to 200 μM had no effect on the macrophage cell viability (Figure 6A). The 24 h.p.i. vvIBDV-infected cell culture medium was then incubated with two concentrations of 4-IPP (50 or 200 μM) for 4 h and then added to macrophages. The mRNA levels of IL-1β (Figure 6B) and IL-18 (Figure 6C) were significantly reduced in the 4-IPP-treated groups compared with the DMSO-treated group.

To further confirm the function of vvIBDV-induced MIF in the transcription of IL-1β and IL-18, MIF inhibitor and anti-MIF antibody were used in the experiments below. Different concentrations (0, 2.5, 5, 10, 20, 40 μM) of ISO-1, iguratimod, or an equal volume of DMSO solvent were added to primary macrophages for CCK-8 experiment to detect the cytotoxicity of ISO-1 and iguratimod (Figure 6D). A total of 20 μM of 4-IPP and 10 μM of ISO-1 and iguratimod were incubated with vvIBDV-infected DT40 supernatant (24 h.p.i.) or equal volume of DMSO (same as 20 μM) for 4 h at RT. Then, the supernatants were added to primary macrophages and maintained for 24 h.p.i. The mRNA levels of IL-1β and IL-18 in macrophages were detected. The results showed that both 4-IPP and iguratimod could inhibit the mRNA level of IL-1β (Figure 6E) and IL-18 (Figure 6F), except ISO-1.

In another experiment, 50 μg/ml anti-MIF antibody (or rabbit IgG) was incubated with vvIBDV-infected DT40 supernatant (24 h.p.i.) for 10 h at 4°C. The supernatants were used to incubate primary macrophages for 24 h.p.i. The mRNA levels of IL-1β and IL-18 in macrophages were detected. As Figures 6E,F described, the anti-MIF antibody was able to inhibit the promotion of IL-1β (Figure 6E) and IL-18 (Figure 6F) mRNA levels by IBDV infection.

Taken together, both chMIF and secreted MIF from vvIBDV-infected bursal cell supernatants could promote the transcription of the proinflammatory cytokines IL-1β and IL-18 in macrophages.

## Discussion

IL-1β and IL-18 are considered to be master cytokines involved in virus-induced inflammatory and adaptive immune responses; for example, in porcine reproductive and respiratory syndrome virus and influenza virus infections (Allen et al., 2009; Ichinohe et al., 2010; Li et al., 2015). IL-1β is a potent pyrogen responsible for the fever caused by pathogens and can induce the upregulation of proinflammatory cytokines (Kanneganti et al., 2006). IL-18 stimulates CD8^+^ and NK cells to participate in the innate immune response (Eldaghayes et al., 2006; Lee et al., 2015). In our study, both IL-1β and IL-18 significantly increased during infection with vvIBDV, which was in accordance with the inflammation detected by histopathological analysis. Therefore, we chose IL-1β and IL-18 for further study on the mechanism underlying the IBDV-induced inflammatory response.

Previous studies demonstrated that the inflammatory response during IBDV infection is mainly manifested as infiltration of inflammatory cells and upregulation of the expression of proinflammatory factors in the BF (Sharma et al., 1989; Ruby et al., 2006; Zhao et al., 2016). A series of studies reported the upregulation of proinflammatory cytokines, such as IL-1β, IL-6, IL-12, IL-8, and IL-18, in vvIBDV infection both *in vitro* and *in vivo* (Stoute et al., 2013; Rasoli et al., 2015; Quan et al., 2017). These results are consistent with those of our study. However, until now, little was known about the mechanism underlying the vvIBDV-induced inflammation. In our iTRAQ assay, we found that the expression level of chMIF increased significantly during vvIBDV infection of primary chicken bursal cells. Further analysis indicated that MIF was required for the induction of inflammation in response to vvIBDV infection.

MIF, discovered in the 1960s, was the first cytokine to be identified (Bloom and Bennett, 1966; David, 1966), and it was named for its inhibition of random migration of macrophages (Bloom and Bennett, 1966; David, 1966). MIF is widely secreted by various cells, such as immune cells, platelets, hepatocytes, and endotheliocytes (Calandra et al., 1994). Activating stimuli can induce the secretion of MIF from preformed cytoplasmic pools, as mediated by Golgi-associated protein p115, which subsequently induces the transcription of MIF to replenish the cytoplasmic pool (Merk et al., 2009). MIF lacks a signal sequence, so the mechanism of MIF release is unconventional. Further study indicated that MIF could be released by secondary neutrophils from stores in the cytosol under conditions of insufficient clearance of apoptotic neutrophils in the sites of infection and autoimmunity (Roth et al., 2015). In addition to mediating acute and chronic inflammatory diseases, MIF is a chemokine ligand that conjugates CXCR2, CXCR4 (Bernhagen et al., 2007), and CXCR7 (Alampour-Rajabi et al., 2015). Thus, leukocyte migration could be trigged by MIF (Bernhagen et al., 2007). As a potent monocyte/macrophage chemoattractant and activator, MIF can upregulate various inflammatory cytokines, such as IL-1, IFNs, and IL-18, as well as the production of reactive oxygen species (ROS) and nitric oxide (NO) (Cunha et al., 1993; Calandra et al., 1994; Santos and Morand, 2009). Furthermore, phagocytosis of macrophages can be promoted by MIF in response to inflammation (Onodera et al., 1997). Evidence indicates that MIF is able to bind the cell membrane receptor CD74 in a CD44-dependent manner, thereby mediating various signaling cascades (e.g., those involving extracellular signal-regulated kinase [ERK]1 and ERK2 activation and nuclear factor kappa B [NF-κB] and Tap63 expression), which results in cell proliferation (Shi et al., 2006). Blocking MIF binding to CD74 could be a pivotal event in regulating monocyte migration and survival during CNS inflammatory responses (Benedek et al., 2013). 4-IPP, a selective MIF inhibitor, could inhibit MIF/CD74 internalization, activate JNK, and dose-dependently inhibit proliferation by inducing apoptosis and mitotic cell death (Varinelli et al., 2015).

Several studies have reported that huMIF plays a pivotal role in pathogenicity and inflammation in Ross river virus (RRV) (Herrero et al., 2011, 2013) and dengue virus (DENV) (Assuncao-Miranda et al., 2010) infection. MIF can induce vascular leakage during DENV infection, thereby promoting hemorrhagic fever (Chuang et al., 2011). During RRV infection, MIF plays an important role in RRV-induced arthritis and causes severe inflammation and tissue damage (Herrero et al., 2011, 2013). Treatment with an MIF antagonist, such as ISO-1, iguratimod (T-614) can ameliorate MIF-dependent inflammatory reactions, namely, the production of proinflammatory cytokines and leukocyte migration, thereby reducing the injury caused by MIF (Al-Abed et al., 2005; Bloom et al., 2016). Since our iTRAQ results showed upregulation of chMIF during vvIBDV infection, we hypothesized that chMIF is engaged in vvIBDV-induced inflammation.

HuMIF inhibits the random migration of inflammatory cells and has been found to stimulate the recruitment of macrophages and other leukocytes (Gregory et al., 2006; Bernhagen et al., 2007). HuMIF has been demonstrated to control inflammatory and atherogenic leukocyte recruitment *via* CXCR2 and CXCR4, and this recruitment also relied on CXCR/CD74 complex. Blocking CD74 inhibits the recruitment of monocytes (Bernhagen et al., 2007). MIF is required for activation of NLRP3 inflammasome independent of its role as a cytokine, and MIF inhibiting could regulate the release of IL-1β and IL-18, confirming that MIF is a critical factor for NLRP3 inflammasome activation in the pathogenic processes (Lang et al., 2018). Recent reports showed that the U1-snRNP could specifically stimulate MIF and IL-1β production in human monocytes and support the upstream regulatory roles for MIF in activating NLRP3 inflammasome and IL-1β production (Shin et al., 2019). 4-IPP, as an MIF inhibitor, can inhibit the reaction between MIF and CD74 by binding to MIF. Blocking the HuMIF/CD74 axis inhibits the MIF-triggered inflammatory effect (Benedek et al., 2013). CD74 is also a receptor for chicken MIF (Kim et al., 2014). Based on the above, we used 4-IPP to confirm if the inflammatory effect was caused by MIF secretion from vvIBDV-infected cell supernatant. Similar with huMIF, chMIF also inhibits the random migration of chicken macrophages (Kim et al., 2010); but whether chMIF has chemokine-like functions similar to those of huMIF is not known. Our study showed that chMIF could induce the migration of PBMCs at 100–5,000 ng/ml, indicating that chMIF has a chemokine-like function. Furthermore, the chMIF and vvIBDV-infected cell supernatants could increase inflammatory cell infiltration, and this phenomenon could be inhibited by selective MIF inhibitor 4-IPP and ISO-1. These results indicate that chMIF secreted from vvIBDV-infected bursal cells initiates infiltration of inflammatory cells. Our data showed that PBMC migration could not be inhibited by iguratimod alone, consistent with previous screening results for MIF inhibitors, but iguratimod might have significant additive effects with glucocorticoids in suppressing IBDV-induced inflammation as Bloom et al. (2016) reported. More details still need to be explored.

Our findings indicated that IL-1β and IL-18 were upregulated by macrophages after treatment with chMIF alone (∼3,000 ng/ml). Another study reported that chMIF alone does not enhance mRNA levels of proinflammatory cytokines (Kim et al., 2010; Park et al., 2016). The differences between these results might be attributable to the different concentrations of MIF used in the experiments. vvIBDV-infected DT40 and bursal cell supernatant could upregulate the mRNA level of macrophage IL-1β/18, and the upregulation could be reduced by treatment with an MIF inhibitor. Additionally, the vvIBDV infection triggered MIF *in vivo* earlier than the upregulation of IL-1β/IL-18 transcription. Besides, primary chicken macrophages could not be effectively infected by vvIBDV (data not shown), suggesting that vvIBDV induced the mRNA upregulation or the cytokines *via* MIF secretion from infected cells. Our study indicated that MIF was upregulated in the early stage of IBDV infection and played a positive role in vvIBDV-induced inflammation. However, the relationship between MIF and viral pathogenicity was still unclear in the current study. The detailed mechanisms of MIF in IBD progression still need to be further explored.

In conclusion, our study demonstrates that chMIF, secreted from vvIBDV-infected target cells, induces inflammatory cell infiltration and production of inflammatory cytokines during the disease progression. This study reports our initial work toward investigating the mechanism underlying IBDV-induced inflammation; further analyses are required to obtain more concrete information regarding this mechanism. Nonetheless, our findings provide sufficient important insights into the mechanism underlying the virus-induced inflammation to be able to consider MIF as a potential therapeutic target to treat IBDV infection.

## Data Availability Statement

The datasets generated for this study are available on request to the corresponding author.

## Ethics Statement

All animal experiments were approved by the Committee on the Ethics of Animal Experiments at the Harbin Veterinary Research Institute (Harbin, China), Chinese Academy of Agricultural Sciences, and performed in accordance with the Guidelines for Experimental Animals of the Ministry of Science and Technology (Beijing, China).

## Author Contributions

AL, HL, and YL performed the experiments. AL, BY, TW, NY, and YW assembled and analyzed the data and prepared the figures. YW, XQ, QW, QP, and XW contributed to the experimental design and interpretation and provided the reagents. YG, LG, and CL analyzed the data. AL, YW, and XW wrote the manuscript. All authors critically reviewed the manuscript.

## Conflict of Interest

The authors declare that the research was conducted in the absence of any commercial or financial relationships that could be construed as a potential conflict of interest.

## Supplementary Material

FIGURE S1 Isolated cells were collected and identified by flow cytometry using mouse anti-chicken monocyte/macrophage antibody KUL01-FITC to evaluate the cell surface expression of markers typically expressed on chicken macrophage by flow cytometry. The purity of macrophages is 92.2%.
