# Supplementary figures and images for: Macrophage Migration Inhibitory Factor Triggers Inflammatory Responses During Very Virulent Infectious Bursal Disease Virus Infection
Source: Front Microbiol. 2019 Oct 1;10:2225. doi: 10.3389/fmicb.2019.02225 (PMC6779731; doi:10.3389/fmicb.2019.02225)

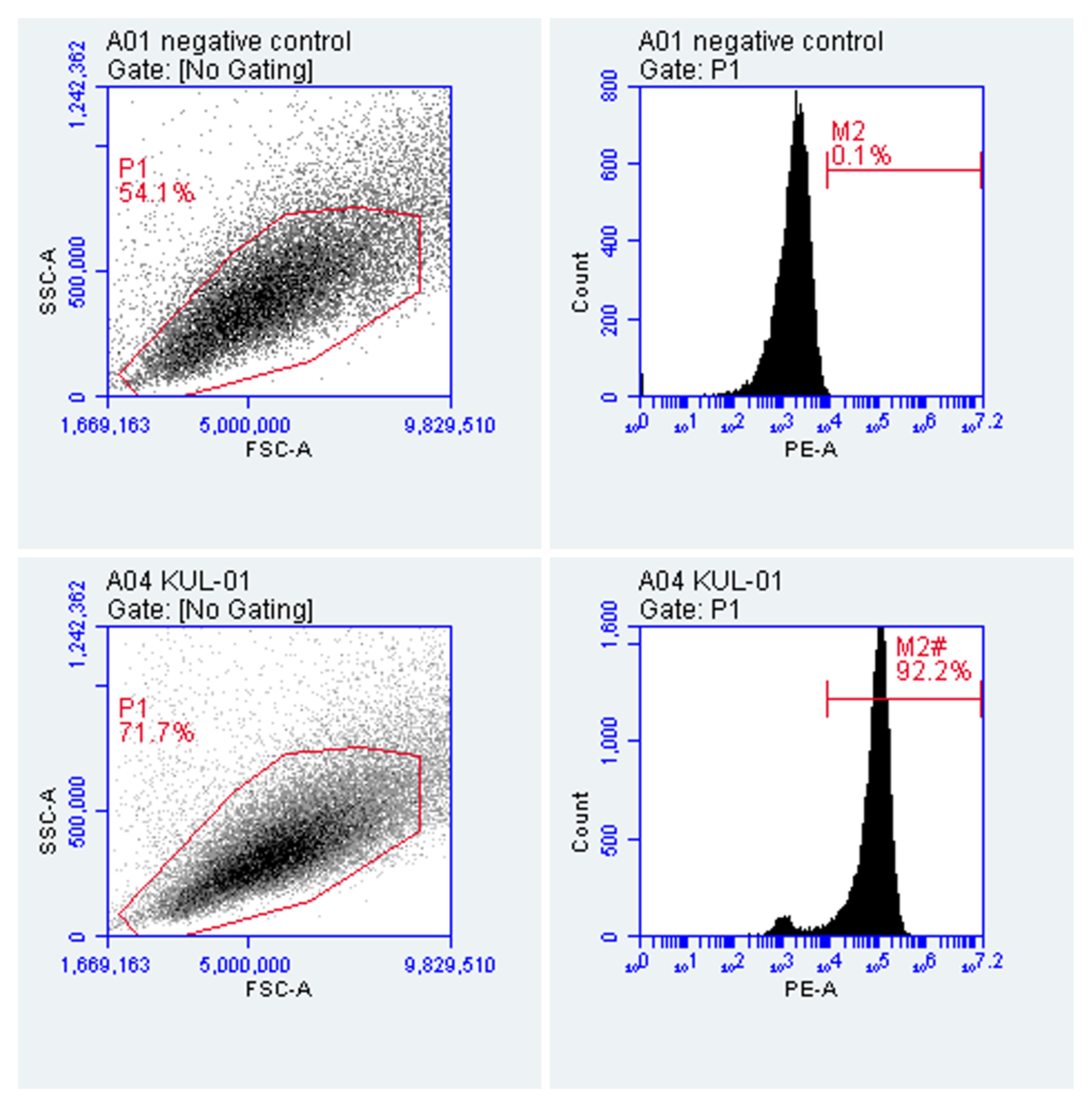

Supplement: FIGURE S1 — Isolated cells were collected and identified by flow cytometry using mouse anti-chicken monocyte/macrophage antibody KUL01-FITC to evaluate the cell surface expression of markers typically expressed on chicken macrophage by flow cytometry. The purity of macrophages is 92.2%. [file Image_1.TIF]
